# Temporal Pattern Analysis of Ultrasound Surveillance Data in Vascular Connective Tissue Disorders

**DOI:** 10.3390/diagnostics14161749

**Published:** 2024-08-12

**Authors:** Corinna Walter, Maria Elisabeth Leinweber, Irene Mlekusch, Afshin Assadian, Amun Georg Hofmann

**Affiliations:** Department of Vascular and Endovascular Surgery, Klinik Ottakring, 1160 Vienna, Austria

**Keywords:** Ehlers–Danlos syndrome, Marfan syndrome, Loeys–Dietz syndrome, connective tissue disease, surveillance

## Abstract

Background: Ehlers–Danlos syndrome (EDS), Marfan syndrome (MFS), and Loeys–Dietz syndrome (LDS) are connective tissue disorders frequently associated with vascular aneurysm formation, dissections, and subsequent major complications. Regular imaging surveillance is recommended for these conditions. However, no guidelines currently exist regarding imaging modality or surveillance intervals. Methods: This retrospective single-center observational study analyzed clinical and imaging data of patients attending an outpatient clinic for vascular connective tissue disorders between August 2008 and January 2024. Imaging (1424 data points in total) and clinical data were extracted from electronic health records. Analysis primarily included a comparison of vessel diameter progression across imaging modalities, with an additional review of the clinical history of vascular events. Results: In total, 19 patients with vascular connective tissue disorders (vCTDs) underwent consultations at our outpatient clinic. Nine (47.4%) patients experienced vascular events, while two (10.5%) passed away during the study period. Multimodal imaging surveillance revealed a tendency towards arterial diameter increase. Consistent ultrasound monitoring provided more reliable diameter progression data for the same arterial segment than a combination of imaging modalities. Temporal analysis indicated a tendency for the continuous growth of the abdominal aorta, the common and internal carotid artery, and the common femoral and popliteal artery. Conclusion: The study highlights the importance of standardized, modality-specific imaging protocols in monitoring patients with vCTDs. The variability in disease progression among these patients further complicates surveillance strategies, contemplating the need for individualized approaches. Further research and prospective multicenter studies are required to refine and improve monitoring protocols.

## 1. Introduction

Several connective tissue disorders (CTDs) affect the vasculature, leading to complications such as true aneurysms or dissections. The three most prevalent pathologies are vascular type Ehlers–Danlos syndrome (vEDS), Marfan syndrome (MFS), and Loeys–Dietz syndrome (LDS). LDS is an autosomal dominant syndrome that is caused by mutations in genes involved in the transforming growth factor beta signaling pathway [1,2]. The most frequently observed clinical triad involves aortic aneurysms with tortuosity, hypertelorism, and bifid uvula or cleft palate [3]. MFS is also an autosomal dominant condition mediated by a mutation in the FBN1 gene, which is responsible for the production of fibrillin [4,5]. MFS presents a variety of signs and symptoms in several different organ systems, but the main reason for decreased life expectancy are aortic dissections or complications arising from aortic root dilation [6]. Ehlers–Danlos syndrome is a heterogeneous group of connective tissue diseases caused by defects in the synthesis of collagen [7,8]. Pathogenic mutations in the COL3A1 gene are associated with vEDS, and patients can suffer from numerous potential complications, including aneurysms up to rupture, arteriovenous fistulas, dissections, and gastrointestinal perforations [9]. Even though all three pathologies feature distinct phenotypic presentations, overlapping cardiovascular signs and symptoms increase the importance of molecular diagnostic confirmation [10,11].

Surveillance and monitoring of the vasculature are frequently proposed for vEDS, either by ultrasound, computed tomography (angiography), or magnetic resonance imaging angiography [9,12]. Patients with MFS are recommended to regularly undergo aortic imaging studies [13,14], and similar recommendations exist for patients with LDS [15,16]. Despite the consensus on the necessity of regular surveillance, precise recommendations or reference standards regarding imaging modalities and the frequency of follow-ups remain undetermined and are often left to the discretion of the respective clinician. This is exacerbated by the lack of body size and age-related normograms for arterial dimensions in patients with vCTDs. A recent survey on the clinical management of vEDS conducted with the members of the Medium-Sized Artery Working Group of the European Reference Network for Rare Vascular Diseases (VASCERN) and additional expert centers confirmed this perception. Although all experts agreed on the importance of monitoring patients with vEDS, no consensus or uniform strategy was found among participating centers, as both screening intervals and modalities used for monitoring diverged [17].

This study aims to evaluate data on imaging surveillance for patients with vascular connective tissue disorders (vCTDs) to contribute to the understanding of disease progression.

## 2. Methods

### 2.1. IRB Approval

The study was approved by the appropriate institutional review board and ethics committee of the City Government of Vienna (ID: EK 23-193-VK). Approval included a waiver of informed consent for this retrospective analysis. The study was conducted according to the Declaration of Helsinki.

### 2.2. Design

This investigation is a retrospective single-center cohort study.

### 2.3. Data

Patients were identified by search queries consisting of “Ehlers–Danlos”, “Marfan”, or “Loeys–Dietz” in the electronic patient documentation. All patient-specific information, such as comorbidities, surgery reports, or follow-up documentation, was extracted from the digital health records. Imaging data were obtained from written reports for all ultrasound studies, or image analyses was conducted by a board-certified vascular surgeon using diameter measurements of computed tomography or magnetic resonance imaging. Ultrasound studies for patients with vCTDs were solely performed using a Logiq S8 device (GE Healthcare, Chicago, IL, USA). Approximately 90% of all ultrasound investigations were conducted by the same operator, minimizing the effects of inter-observer variances (however, intra-observed differences were still present).

This analysis focuses on arterial territories accessible to ultrasound studies, with the following segments being considered for the analysis: internal carotid, external carotid, common carotid, abdominal aorta, common iliac, internal iliac, external iliac, common femoral, and popliteal arteries. In summary, 1424 data points were extracted and analyzed.

### 2.4. Analysis

Apart from patients with genetically confirmed vCTDs, multiple patients attended the outpatient clinic for vascular connective tissue disorders for preliminary consultations but did not present associated genotypes in subsequent diagnostic tests. These patients were used as controls to compare the imaging data of patients with vCTDs. The analysis featured a multi-layered approach. First, data from all imaging studies (ultrasound, computed tomography, and magnetic resonance imaging) available in the electronic health records were extracted and chronologically assessed. Relative diameter progressions between the first and last available imaging studies were calculated for each patient and available segment, with >10% increase considered relevant. A comparative analysis between diameter progressions in patients with vCTDs and controls was conducted. Subsequently, only ultrasound studies in patients with confirmed vCTDs were analyzed to assess relevant progression. Temporal patterns were assessed by aggregating data points from the study population either based on time under surveillance or patient age at point of measurement.

Patient characteristics were analyzed by descriptive statistical methods, including calculation of measure of central tendency and dispersion. All statistical analyses were performed with R version 4.1.3 (R Foundation for Statistical Computing, Vienna, Austria) in RStudio (Posit PBC, Boston, MA, USA).

## 3. Results

### 3.1. Sample Characteristics

From August 2008 to January 2024, 34 patients underwent consultations at the outpatient clinic for vCTDs. Subsequent genetic tests revealed no vCTD in 15 patients, while 13 had verified vEDS, 3 had LDS, and 3 had MFS. The mean age at the first available imaging study was 32.5 years. Six patients had medically treated arterial hypertension, nine had a history of cardiovascular surgery (three prior and six after initiating surveillance at our center), three had a history of aneurysm rupture (with one occurrence prior to and two during follow-up), and five had a history of arterial dissection (with four prior to and one during follow-up). Aneurysm ruptures occurred in the posterior tibial artery, the internal carotid artery, and the splenic artery, whereas dissections were found in the ascending and descending aortas, common and internal carotid arteries, and the renal, celiac, vertebral, external iliac, common femoral, and left anterior descending arteries. Four of five patients with arterial dissections had multiple affected arterial territories. Clinical information stratified by underlying pathology is shown in Table 1. Two patients (10.5%) died during the observational period due to vascular complications of their underlying vCTD, i.e., one dissection with subsequent bleeding of the renal artery and one rupture of the splenic artery. The longest surveillance period, defined as the time between the first and last available imaging study, was 3008 days (8.2 years). The study population was significantly heterogeneous, with arterial complications occurring in various territories including intracranial, aortic, visceral, and peripheral arteries. However, there was a tendency of complications clustering in individual patients, as six patients (mean age: 32.8 ± 9.9 years) had multiple events (including surgery, dissection, or rupture), whereas nine patients (mean age: 26.8 ± 9.6 years) did not have any arterial complications so far. Arterial progression scores [18] were also calculated for patients with vEDS. Seven patients remained stable at 0 during the monitoring period, whereas two patients with the largest increases and absolute values (from 21 to 33 and from 0 to 12, respectively) died due to arterial complications.

### 3.2. Combining Imaging Modalities

As the first step in the multi-layered analysis plan, diameter growth in arterial segments between the first and last available imaging studies was investigated and compared between patients with vCTDs and controls. Some limitations were observed, i.e., when combining different imaging modalities for the same arterial segment, diverging measurements with decreasing vessel diameters over time were noted. This finding was more frequent in controls than patients with vCTDs (Table 2). Surveillance periods varied profoundly between patients and the limited number of available controls (Supplementary Table S1).

Nevertheless, most arterial segments showed a tendency towards increasing size (minimum growth of 10% during the observational period) in patients with vCTDs, with the common femoral artery and the abdominal aorta showing growth in 50.0% and 40.0% of cases, respectively.

### 3.3. Consecutive Ultrasound Studies

The subsequent step of the analysis plan featured only data points resulting from patients with verified vCTD undergoing ultrasound surveillance at our outpatient clinic (*n* = 12). Decreasing vessel diameters were still recorded in multiple patients and arterial territories but to a lesser extent than in the previous analysis step. Focusing solely on ultrasound investigations leads to more homogenous results, with vessel diameters mainly increasing in most segments. Analogous to the first analysis, the abdominal aorta was most prominently affected, followed by the common femoral and popliteal arteries. Diverging results were prominently found in the common iliac artery (Table 3). Only two patients had no relevant growth in any of the 17 studied arterial segments. However, those patients had surveillance periods of 166 and 1040 days. One patient (vEDS) showed growth in 13 territories (monitoring period: 2537 days), while two patients (both LDS) had increasing diameters in eight segments (monitoring periods: 2691 and 2440 days). Taking into account the limited sample size, there was a tendency towards a positive association of arterial segments with documented diameter growth with increasing age and time under surveillance (Supplementary Figure S1).

In this study population, two patients developed bilateral internal carotid aneurysms (both vEDS), while one patient developed bilateral popliteal artery aneurysms (LDS). Surveillance periods were 1317 and 2537 days (starting at ages 28 and 49 years, respectively) for the internal carotid aneurysms and 2286 days (starting at age 29) for the popliteal artery aneurysms.

### 3.4. Temporal Patterns

**Figure 1:**
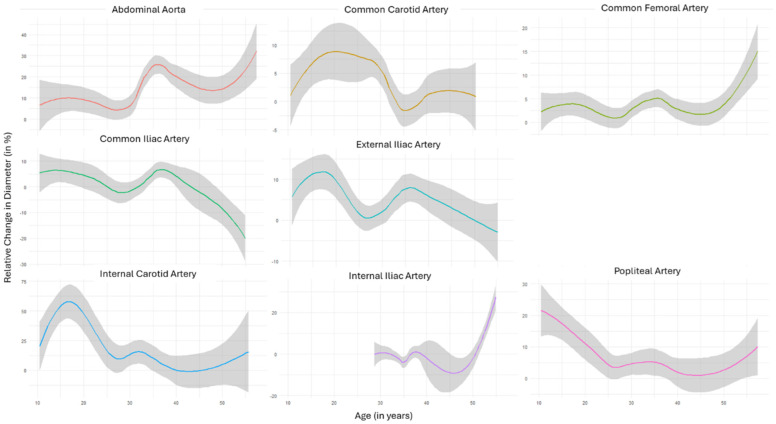
Aggregated trendlines represent the relative change in diameter (in percent) plotted against the age of the study population of different segments. Shaded gray area reflects 95%CI.

To investigate the temporal patterns of arterial diameters in different segments and thus the natural disease progression, the available data points were aggregated and plotted as trendlines. Investigating relative growth in arterial diameters at various ages (Figure 1) showed that the abdominal aorta, the common and internal carotid arteries, and the common femoral and popliteal arteries had a tendency for continuous growth.

**Figure 2:**
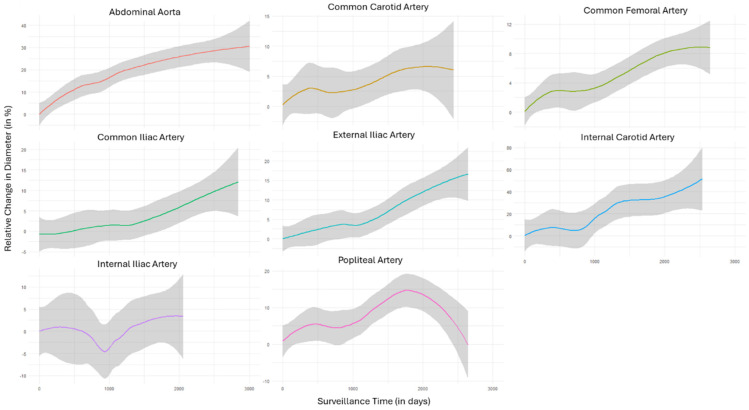
Aggregated trendlines represent the relative change in diameter (in percent) plotted against the surveillance period of the study population of different segments. Shaded gray area reflects 95%CI.

**Figure 3:**
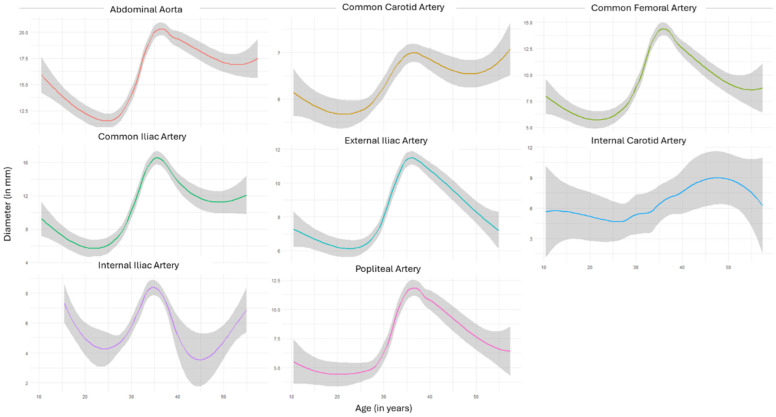
Aggregated trendlines represent artery diameters (in mm) plotted against the age (in years) of the study population of different segments. Shaded gray area reflects 95%CI.

Measurements from the iliac arteries showed a less homogenous picture, reflecting the decreasing artery diameters documented in several patients over the surveillance period (Table 2). To account for the effect of time under surveillance, aggregated trendlines were analogously plotted with respect to the surveillance time (Figure 2). Except for the popliteal artery, the aggregated data of all territories showed continuously increasing diameters over the surveillance period. Finally, the data aggregation of absolute diameters over different ages showed that in almost all territories, arterial diameters peaked between the ages of 30 and 40 (Figure 3).

## 4. Discussion

From August 2008 to January 2024, 34 patients were examined at the outpatient clinic for vCTDs. Genetic testing confirmed vEDS in 13 patients, LDS in 3 patients, and MFS in 3 patients, while 15 patients showed no evidence of vCTDs. Their demographic and clinical profiles as well as disease progression resembled larger observational cohorts affected by vCTDs [18,19]. The first step in the multi-layered analysis involved comparing diameter growth in arterial segments between patients with vCTDs and controls using different imaging modalities. Results indicated that combining modalities often led to diverging measurements, with sporadically decreasing vessel diameters over time, reflecting the difficulty of comparing ultrasound and computed tomography measurements. Despite this variability, most arterial segments in patients with vCTDs exhibited a tendency for size increase, with the common femoral artery and abdominal aorta showing growth in 50% and 40% of cases, respectively. Focusing solely on ultrasound vessel mappings in patients with vCTDs resulted in more homogenous measurements, indicating that committing to a single imaging modality for a specific arterial segment is more reliable in a continuous surveillance program.

In the temporal analysis to study the natural course of vCTDs, we found decreasing growth rates and vessel diameters after the age of 40. This likely means that patients with vCTDs surpassing that age experience less aggressive manifestations, whereas patients with faster-progressing pathologies experience highly aggressive manifestations in their twenties and thirties with subsequent arterial complications.

Imaging surveillance is crucial for managing patients with vascular connective tissue disorders such as Marfan syndrome, Ehlers–Danlos syndrome, and Loeys–Dietz syndrome. These conditions predispose individuals to aortic and arterial aneurysms, dissections, and ruptures, necessitating regular monitoring to mitigate life-threatening complications. Despite consensus on the necessity of surveillance, optimizing the intervals between imaging studies remains a complex challenge. For example, the 2018 clinical practice guidelines on EDS consider optimizing surveillance and treatment decisions as one of the key challenges for further research [20]. The frequency of imaging must balance the need for the timely detection of pathological changes against the risks and burdens of repeated imaging.

The primary imaging modalities used for surveillance include magnetic resonance angiography (MRA), computed tomography angiography (CTA), and ultrasound. Each modality has distinct advantages and disadvantages. MRA offers high-resolution images without ionizing radiation and superior soft tissue contrast, which can be beneficial for visualizing the aortic wall but is less widely available, more costly than CTA, and not applicable to certain patients [21,22].

CTA provides high spatial resolution and rapid acquisition times, making it excellent for visualizing vascular anatomy and calcifications [23]. Nevertheless, the risks associated with repeated radiation exposure make CTA less optimal for long-term surveillance, particularly in young patients [24].

Ultrasound, while free of ionizing radiation and generally does not require contrast agents, is operator-dependent with variable reproducibility [25,26]. It is portable and relatively inexpensive [27], making it useful for initial screening and follow-up of superficial vessels. However, its utility is limited by patient body habitus and the difficulty of visualizing deep or complex arterial territories, such as the thoracic aorta and intracranial vessels. Additionally, ultrasound provides inferior resolution compared to MRA and CTA for detailed vascular imaging [28]. Nevertheless, arterial segments that are accessible to ultrasound studies comprise 50–70% of territories with vascular complications in patients with vEDS [29]. Peripheral aneurysms are also present in approximately a third of patients with MFS and are additionally associated with advanced aortic pathologies [30]. While our surveillance program focusses on peripheral arteries, echocardiography further expands opportunities of ultrasound-based monitoring by providing valuable insights into pathologies of the ascending aorta. Future surveillance protocols might combine imaging modalities to counterbalance trade-offs in accessibility, invasiveness, and need.

As illustrated by the results of this study and previously reported issues in other settings such as conventional abdominal aortic surveillance [31,32], combining different imaging modalities can lead to implausible and potentially hazardous conclusions. Even when committing to a single imaging modality, reproducibility remains a challenge due to inter- and/or intra-observer variability or patient factors such as weight and blood pressure alterations [33]. The standardization of imaging protocols, including the documentation of the exact point of measurement of a given vessel diameter, is essential when implementing a surveillance program for patients with vCTDs. Variations in diameter measurements in the iliac arteries in the results of this study are likely attributable to varying points and plains of measurement.

Multiple patients showed an increase in diameter of the abdominal aorta. Vascular CTDs are highly associated with both aneurysms and dissections of the aorta and contribute significantly to the high mortality and limited life expectancy of affected patients [34,35]. The 2022 ACC/AHA guidelines for the diagnosis and management of aortic disease therefore recommend the regular imaging of the aorta for MFS, LDS, and vEDS. However, concrete evidence and strong corresponding recommendations primarily exist for transthoracic echocardiograms [14]. This reflects the scarce evidence for further aortic surveillance and need for additional investigations regarding optimal surveillance imaging modalities and intervals. We should refrain from simply extrapolating results and recommendations for patients with aneurysm that are not affected by vCTDs as these patients frequently suffer from aortic complications at diameters that commonly do not lead to ruptures in patients without vCTDs [11]. Taking all this into consideration, annual ultrasound investigations of the abdominal aorta might be favored to CT- or MRI-based imaging every 3–5 years that is discussed in certain guidelines.

Nevertheless, multiple gaps in the evidence persist for these patients. Determining the optimal interval between studies is particularly difficult. Imaging too frequently, particularly in clinically silent patients, can lead to unnecessary exposure to ionizing radiation, especially with CT angiography, waste resources, and can affect a patient’s lifestyle and overall well-being, while infrequent imaging may miss critical changes in vessel dimensions or morphology. The current guidelines often provide broad recommendations, but individual patient factors, such as the specific genetic mutation, family history, and previous aortic dimensions and growth rates, necessitate a tailored approach. The natural history of these disorders varies significantly, with some patients experiencing rapid progression while others remain stable over longer periods, complicating the establishment of universal surveillance protocols. Another significant challenge in imaging surveillance for these patients is the difficulty in associating growth rates and absolute vessel diameters with rupture or complication rates. Unlike patients with typical aneurysm, patients with vCTDs often exhibit different patterns of disease progression. For instance, the rapid growth or significant enlargement of a vessel does not show a linear correlation with arterial complications, making it challenging to predict adverse outcomes based on imaging findings alone.

This study has several limitations that need to be acknowledged. Firstly, its retrospective nature inherently introduces potential biases. The data were collected from past records, which may be incomplete or inconsistent, impacting the accuracy and reliability of our findings. Secondly, the study was conducted at a single center, which may limit the generalizability of the results to broader populations. The specific practices, patient demographics, and clinical approaches of our center may not reflect those of other institutions. Additionally, the small patient cohort further restricts the robustness of our conclusions. Due to the limited number of cases, the findings, in general, and the affected arterial segments as well as growth rates, in specific, should be interpreted with caution. Lastly, the lack of standardization in the performed imaging studies adds another layer of variability. These limitations highlight the need for larger, multicenter, prospective studies with standardized imaging protocols to validate and extend our findings.

## 5. Conclusions

In conclusion, while imaging surveillance is essential for managing patients with vascular connective tissue disorders, optimizing the intervals between studies and choosing the appropriate imaging modality involves careful consideration of the trade-offs between accuracy, reproducibility, and the risks associated with each technique. The variability in disease progression among these patients further complicates surveillance strategies, highlighting the need for individualized approaches and the potential for ongoing research to refine and improve monitoring protocols. Furthermore, it will require large longitudinal imaging datasets to either deduce average growth rates and vessel diameters and extrapolate natural disease progression or conclude that the course of vCTDs is too variable and patient specific. Currently, it is difficult to quantify a patient’s individual risk for arterial complications in the absence of conclusive references that illustrate the temporal patterns of vCTDs. While there is a need for prospective multicenter studies to define precise guidelines, data such as those generated in this study provide valuable insights regarding disease progression to assist in designing future investigations.

## Figures and Tables

**Table 1:** Baseline characteristics of the study population. Age at the first imaging study is given as the arithmetic mean (sd) for Ehlers–Danlos syndrome and in total in years. The ages of the patients at the first imaging study with Loeys–Dietz and Marfan syndromes are presented in years.

	Ehlers–Danlos	Loeys–Dietz	Marfan	Total
Number	13	3	3	19
Age	31.4 (10.9)	29.6/31.7/38.7	24.0/33.5/52.2	32.5 (9.6)
Arterial Hypertension	4 (30.8%)	0 (0%)	2 (66.7%)	6 (31.6%)
History of cardiovascular surgery	5 (38.5%)	1 (33.3%)	2 (66.7%)	8 (42.1%)
History of aneurysm rupture	3 (23.1%)	0 (0%)	0 (0%)	3 (15.8%)
History of arterial dissection	3 (23.1%)	0 (0%)	2 (66.7%)	5 (26.3%)

**Table 2:** Number of vessels with a diameter increase or decrease of at least 10% in the given arterial segment.

Territory	Increase		Decrease	
	Patients	Controls	Patients	Controls
Common Carotid	8 (36.4%)	0 (0%)	1 (5.6%)	4 (66.7%)
Internal Carotid	6 (30.0%)	0 (0%)	3 (18.8%)	2 (50.0%)
External Carotid	2 (33.3%)	0 (0%)	0 (0%)	1 (50.0%)
Abdominal Aorta	6 (40.0%)	1 (20.0%)	0 (0%)	1 (20.0%)
Common Iliac	5 (16.7%)	3 (37.5%)	5 (16.7%)	1 (12.5%)
Internal Iliac	7 (35.0%)	1 (16.7%)	0 (0%)	1 (16.7%)
External Iliac	11 (36.7%)	1 (12.5%)	4 (15.4%)	1 (12.5%)
Common Femoral	12 (50.0%)	4 (66.7%)	3 (12.5%)	0 (0%)
Popliteal	7 (31.8%)	1 (50.0%)	2 (9.1%)	(0%)

**Table 3:** The number of vessels with a diameter increase or decrease of at least 10% in the given arterial segment in patients with a connective tissue disease based on only ultrasound studies.

Territory	Increase	Decrease
Common Carotid	3 (15.0%)	0 (0%)
Internal Carotid	6 (30.0%)	1 (5.0%)
External Carotid	0 (0%)	0 (0%)
Abdominal Aorta	7 (58.3%)	0 (0%)
Common Iliac	4 (20.0%)	3 (15.0%)
Internal Iliac	1 (12.5%)	0 (0%)
External Iliac	4 (20.0%)	1 (5.0%)
Common Femoral	8 (42.1%)	0 (0%)
Popliteal	8 (36.4%)	2 (9.1%)

## Data Availability

Data can be made available upon reasonable request.

## Supplementary Materials

The following supporting information can be downloaded at: https://www.mdpi.com/article/10.3390/diagnostics14161749/s1, Table S1. Surveillance periods given a median (Q1–Q3) per arterial segment in days; Figure S1. Bubble plot illustrating the relationship between age, time under surveil-lance and number of enlarging arterial segments (bubble diameter).
