# Supplementary material for: Temporal Pattern Analysis of Ultrasound Surveillance Data in Vascular Connective Tissue Disorders
Source: Diagnostics (Basel). 2024 Aug 12;14(16):1749. doi: 10.3390/diagnostics14161749 (PMC11354072; doi:10.3390/diagnostics14161749)

Supplementary Table S1. Surveillance periods given a median (Q1 – Q3) per arterial segment in days. If less than 4 data points were available, the individual data points are depicted separated by slash.

Territory	Patients	Controls
**Common Carotid**	1283 (1037 – 2280.75)	153 / 329
**Internal Carotid**	1317 (1040 – 2236)	153 / 329
**External Carotid**	734 (730 – 1232)	153
**Abdominal Aorta**	1298.5 (392.5 – 2595.5)	883 (189 – 1442)
**Common Iliac**	1028.5 (383.75 – 2374.25)	568.5 (231.75 – 1113)
**Internal Iliac**	1283 (464.25 – 1997.5)	189 / 613 / 883
**External Iliac**	1028.5 (524 – 2561.5)	568.5 (237.75 – 1113)
**Common Femoral**	1017 (397 – 2440.5)	189 / 883 / 2537
**Popliteal**	2044.5 (1109.25 – 2440.75)	-

Supplementary Figure S1. Bubble plot illustrating the relationship between age, time under surveillance and number of enlarging arterial segments (bubble diameter). Two patients without growing vessel diameters are not shown.

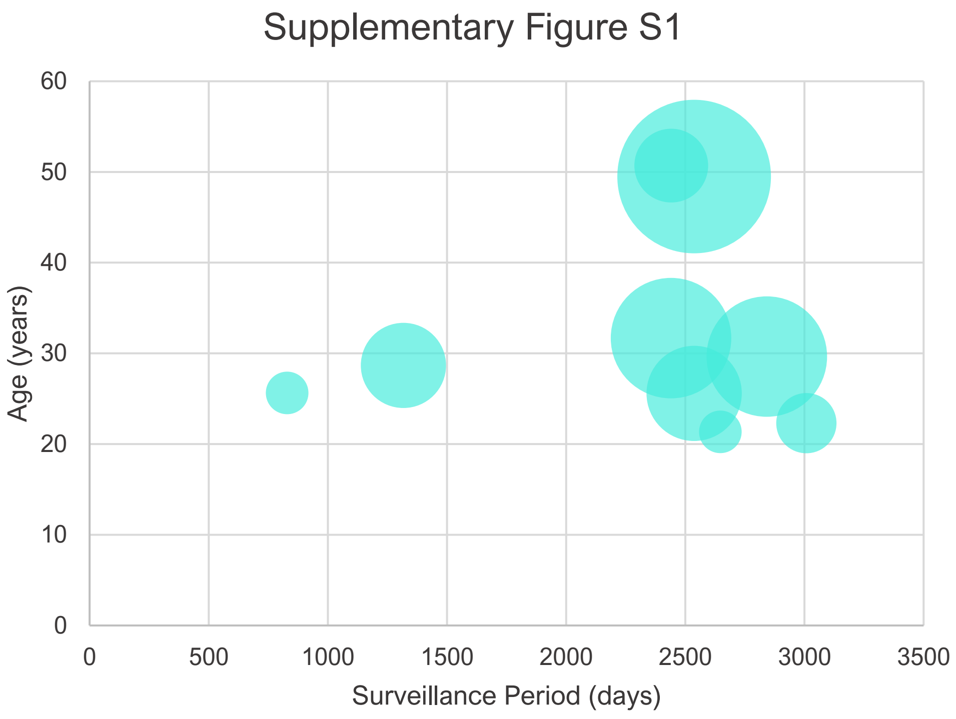

Supplement: Supplementary file 1 [file diagnostics-14-01749-s001.zip › diagnostics-3105225-supplementary.pdf]
